# Nationalism, conspiracy theories and vaccine mandates: Exploring the statism determinants for attitudes to COVID-19 control in China

**DOI:** 10.1016/j.jvacx.2023.100263

**Published:** 2023-01-18

**Authors:** Ruifen Zhang, Jun Yan, Hepeng Jia, Xi Luo, Jingke Lin, Qinliang Liu

**Affiliations:** a School of Communication, Soochow University, Suzhou 215127, China; b School of Journalism and Information Communication, Huazhong University of Science and Technology, Wuhan 430074, China; c School of Public Health, Soochow University, Suzhou 215127, China; d School of Journalism and Communication, Sun Yat-sen University, Guangzhou 510006, China

**Keywords:** COVID-19, Vaccination, Nationalism, Conspiracy theories, Mandatory vaccination, Statism

## Abstract

**Introduction:**

China’s loosening its COVID-19 controls highlighted its insufficiency in vaccination protection. Mandatory vaccination might be necessary if the gap cannot be filled over a short time. However, few studies have explored how Chinese people view the COVID-19 vaccine mandates, let alone placing such views in the country’s highly politicized context.

**Material and methods:**

The current study utilizes data from a national survey adopting quota sampling to analyze the Chinese public's medical and non-medical considerations when judging compulsory COVID-19 vaccination (n = 1,523). The survey was conducted between 1 and 8 April 2021. All adults aged 18 years and older were eligible to take part. The survey included sociodemographic details, perceived susceptibility to infection, perceived vaccine benefit, attitudes to vaccination policies, nationalism, beliefs in various conspiracy theories and science literacy. Multiple regression analyses were done to examine factors associated with the attitude to COVID-19 vaccine mandates.

**Results:**

The study reveals that personal risk and benefit perceptions did not dominate the Chinese public’s attitude toward vaccination mandates. Instead, nationalism was relatively strongly associated with their willingness to accept mandatory vaccination. Contrary to studies in the West, various conspiracy beliefs and conspiratorial thinking were robustly related to the support for mandatory vacciniation. Science literacy didn’t link to the attitude to vaccination mandates. It only had a weak moderating effect on the influence of conspiratorial thinking on attitudes to the vaccination policies.

**Conclusions:**

The results indicated that Chinese people’s attitude to the COVID-19 vaccination policy is highly politicized and influenced by conspiracy theories. Given the potentially massive impacts of the COVID-19 infection, we need to educate the Chinese public with more medically valuable and relevant information to help them make sound decisions regarding vaccination. Meanwhile, we can adopt nationalistic tones to improve the persuasion effect, but misinformation during the process must be overcome.

## Introduction

After three years of the desperate struggle to maintain zero-COVID, China relaxed COVID restrictions in early December 2022, which, though highly welcomed by the public, has witnessed a wave of infections [1]. While the country’s strict lockdown measures had avoided many infections and deaths [2], it kept the herd immunity level low among average Chinese. To cope with the impact, China launched new rounds of vaccination against the pandemic [3].

Fighting the pandemic urgently needs a dramatic increase in vaccination coverage [4]. Vaccine mandates may still be needed in China even though the policy has been abandoned by many nations. Improving effective communication around vaccination requires us to understand better the factors underlying people's attitudes to mandatory vaccination. Large amounts of studies have examined such attitudes and their determinants. They covered demographic features, personal perceptions (risk perception, felt health benefits, and judgment of vaccine efficacy, etc.), political and partisan influences, ethical aspects, and intervention measures to improve the acceptance of mandatory vaccination [5], [6], [7].

In the Chinese context, while the abovementioned and more studies have explored people’s intention for COVID-19 vaccination and immunization policies [8], [9], [10], most failed to explore how China’s highly politicized environment for COVID-19 control may have impacted people’s decisions and opinions [11]. To fill the gap, the current study tries to employ people’s political beliefs and their acceptance of conspiracy theories, which have been found to closely link to political stance [12], [13], to measure the public attitude to China’s vaccination policies. We believe as vaccination has been increasingly politicized worldwide [14], [15], the current study can broaden our understanding of people’s attitudes to vaccines and their determinants well beyond COVID-19 and China contexts.

## Theoretical reasoning

### Demographic and risk factors associated with COVID-19 vaccination attitude

While our central interest is to reveal the politicized determinants for people’s attitudes to COVID-19 vaccination policies, we place this consideration into a set of regular factors associated with the public views about vaccinations. Among them, demographic factors including older age, female and higher income have been found to influence people’s attitude to vaccination mandate [16], [17], [18], [19], [20]. Besides, risk perceptions were widely researched. People with a high perception of risks – in terms of both vulnerability to disease and the perceived severity – were more desirable of taking the jabs and relatively more supportive of vaccine mandates [21], [22], [23]. Similarly, the perceived benefit of COVID-19 vaccines boosted people’s attitude to and intention for personal vaccination and mandatory vaccination [24], while the lack of benefit feeling caused vaccine hesitancy [19]. Due to the huge inconvenience that the forcible quarantine and blockade taken by China have caused to infected individuals during COVID-19, it is also necessary to examine the role of the perceived risk of being quarantined in influencing people’s attitude to mandatory vaccination. Taking these factors together, we should expect risk and benefit perceptions to be placed among determinants of the Chinese public’s attitude to compulsory vaccination. Therefore, this paper proposes the following research hypotheses:

H1 The more the participants perceived they were susceptible to the COVID-19 infection, the more likely they would support COVID-19 mandatory vaccination.
H2 The higher severity of infection the participants perceived, the more likely they would support COVID-19 mandatory vaccination.
H3 The higher the participants perceived the benefit of vaccination against COVID-19, the more likely they would support mandatory vaccination.
H4 The higher the participants perceived the risk of being quarantined, the more likely they would support the COVID-19 mandatory vaccination.

### Nationalism and conspiracy theories associated with COVID-19 vaccination attitude

Several studies have shown that partisanship influences the public’s attitude toward mandatory vaccination. In the United States, for example, liberals were more likely to support mandatory vaccination[25]. Though the directions of support by partisans were inconsistent across nations [26], political inclinations were strong predictors of attitudes to mandatory vaccination in many Western countries [27]. In China, political polarization and its influences have become increasingly apparent [28], [29]. Scholars often used nationalism as a proxy measure to examine the health consequences of the people’s sociopolitical values [30], [31], [32]. To follow this trend, this study will probe the effect of people’s nationalism score on their attitude to COVID-19 vaccine mandates.

Indeed, in the COVID-19 setting, a multi-country (67 countries) study reported that stronger national identity – the degree for people to identify with their nation – was consistently associated with greater engagement in public health behaviors and support for public health policies [33]. The study also found that higher levels of national identification before the pandemic predicted lower mobility during the early stage of the pandemic (r =  − 0.40) [33]. The reason is simple. The national governments and their affiliated experts implement the pandemic control, actively distribute risk information, and encourage public health participation. Nationalism evaluates the degree of national identity and the latter can be considered a specific form of nationalism [34]. Therefore, we hypothesize the following statement:

H5 The higher nationalism level the participants had, the more likely they would support the COVID-19 mandatory vaccination.

**Conspiracy theories and scientific knowledge associated with COVID-19 vaccination attitude**

The public’s politicized rejection of mandatory vaccination is often embodied in conspiracy beliefs [17]. In the Chinese setting, a higher level of nationalism was associated with the more enthusiastic embrace of outgroup conspiracy theories regarding COVID-19 – those claiming that the SARS-CoV-2, the virus that causes the pandemic, first appeared outside China or was manufactured against China [35], [36]. A recent study found that users who engaged in discussions about COVID-19 conspiracies on Chinese Weibo used more national identity expressions in everyday social media conversations [37].

Conspiracy theories shake the authority of scientists and the government hiring them [38]. Therefore, believing the pandemic is a conspiracy naturally leads to rejecting mandatory vaccination [39]. Hence, we hypothesize:

H6 The more the participants believed in COVID-19 and vaccine-related conspiracy theories, the less likely they would support COVID-19′s mandatory vaccination.

However, different conspiracy theories may result in divergent consequences in health behaviors in the COVID-19 setting [35], [40]. Correspondingly, scholars adopted conspiratorial thinking, the sensitivity to conspiracy-based explanations of social and political phenomena, to examine the wide impact of irrational belief [41]. Recent studies have also identified conspiratorial thinking was negatively related to the COVID-19 vaccine [42], [43]. Conspiratorial thinking, also called conspiracy mentality, typically measures people’s acceptance of the incomprehensibility of the actions of the government, companies, and elites [44]. In the COVID-19 setting, conspiratorial thinking predicted the rejection of vaccination against COVID-19 [45], so we should expect that among the Chinese respondents, such conspiratorial thinking should negatively impact their attitude towards mandatory vaccination policy.

H7 The stronger conspiratorial thinking the participants had, the less likely they would support the mandatory vaccination of COVID-19.

In studies related to conspiracy theories, scholars often examine whether science literacy – a measure used to assess both scientific knowledge and analytical thinking – can reduce conspiracy belief and its consequences [30], [31]. For COVID-19 vaccination, vaccine knowledge has been found to correlate positively with people’s vaccine uptake against the pandemic [46]. Then, in examining political and conspiracy impacts on Chinese people's attitude to mandatory vaccination, what role may science literacy have played? We propose the following hypothesis and research question:

H8 The higher the science literacy level the participants had, the more likely they would support mandatory vaccination of COVID-19.

RQ What role did science literacy play in the relationships between conspiracy theory beliefs, conspiratorial thinking, and the attitude to mandatory inoculation of COVID-19?

## Materials and research methods

### Research design

We commissioned a Shanghai-based research company to conduct an online survey between the 1st and 8th April 2021. The sample was collected from all 31 provinces and municipalities in China with quota sampling in accordance with the Statistical Yearbook 2019 of China. The school that hired the main researchers conducting the survey belonged to a large public university in eastern China. Like many other Chinese universities, there wasn’t an institutional review board for social science research at the university. Instead, we sought the permission from the school leadership after reporting the details of our study design, potential risks informants might face and our protection measures.

Although full vaccination coverage (two doses) has reached 90% so far, China may need a new round of booster vaccination to square up to the huge impact of the pandemic after its recent abandoning of zero-COVID policies [47]. Besides, conspiracy theories ranging from the foreign origins of COVID-19 and US entities’ synthesis of the virus remained popular on Chinese social media when this paper was written [48]. Therefore, we don’t think our data are outdated. Instead, it is still relevant in revealing common determinants underlying the Chinese public's attitude to vaccine mandates.

Participants filled in the questionnaire anonymously. They were informed that they could leave at any time without telling the reason. We obtained the demographic data of each participant, including age, gender, educational level, monthly income, and whether he/she was vaccinated. Data shows that the COVID-19 vaccine coverage in China was 9.72% when the questionnaire was issued [49], but our questionnaire shows that 7.26% of the participants have been vaccinated. Therefore, we managed the sample in accordance with this ratio to make it conform to the actual situation in China. According to the researcher's pre-test, the final reliability of the questionnaire was 0.92.

### Measurement scales

To examine participants’ attitudes towards mandatory vaccination against COVID-19, we asked them to what extent they agreed with four statements, including the attitude to the vaccination’s impact on individual freedom, the jurisdiction of the government in managing quarantine and lockdowns, and the public's right to make their own travel choices. The concrete questions for this and other variables examined in this study were provided in the Appendix. Replies to the statements had accepted internal consistency (Cronbach’s α = 0.70). The mean value of the four questions is taken as the final score of the attitudes toward mandatory vaccination.

Next, we asked participants to answer six questions to measure the level of nationalism. We used an updated instrument by expanding a well-established measure to examine nationalism [30], [50]. The measure consisted of six questions (Cronbach’s alpha = 0.86). The former three questions measured the level of public support for the state and government in general, and the latter three were explicitly designed for the COVID-19 situation, measuring their confidence in China's performance during the pandemic. The score of the nationalism level is the mean of all six questions.

Then, respondents were also asked how much they agreed with popular conspiracy theories. The measure consisted of four questions about beliefs in COVID-19-related and vaccine-related conspiracy theories, involving the origins of COVID-19 (from a foreign military lab and a Chinese lab, respectively), the effort to hide negative consequences of the COVID-19 vaccine, 5G technology’s supportive role for the spread of the virus. Cronbach’s alpha for the scale was 0.74, representing good internal consistency. The score of the conspiracy belief is the mean of all questions.

We also set five questions, which were widely used in previously published research [42], [45], [51], to measure conspiratorial thinking. These five questions (Cronbach’s alpha = 0.90) were as follows: 1) The inside story of many essential things in the world is kept from the public; 2) Officials often do not tell us about the real motives behind their decisions; 3) Government agencies closely monitor all citizens; 4) Incidents that appear to be unconnected are often the result of covert activities; 5) Some secret organizations have a significant influence on political decision-making. The score of this variable is the equivalent of the mean value of the above questions.

Finally, we measured participants’ science literacy (Cronbach’s alpha = 0.83), using 11 yes/no and multiple-choice questions to examine participants' familiarity with scientific procedures, their analytical thinking, and their basic scientific knowledge, respectively. The questions were adopted from a well-established, globally used instrument [52]. One point was added to each correct answer, and no point was added to the wrong and “I don’t know” answers.

Except for nationalism (7 points Likert) and science literacy, all other variables were measured on a five-point scale. Participants chose the number that best matched their ideas as the answer in accordance with the questionnaire prompt.

### Statistical analysis

We first used descriptive statistics to analyze demographic information, perceptions of risks and benefits, science literacy, nationalism, conspiracy theory beliefs and conspiratorial thinking. Then, we used multiple linear regression to analyze the relationship between the attitude to mandatory vaccination of the Chinese public and the other factors mentioned above. The software used for statistical analysis is SPSS Version 25.0, and the confidence level is set to be 95%.

## Results

### Demographic characteristics

We received 2,038 completed responses. As mentioned above, we matched the vaccination variable against the actual situation (9.7%) and finally got 1,523 valid questionnaires. Table 1 showed the demographic breakdown of the groups. Over 51.5% of the participants were men and the rest were women. Most respondents were middle-aged (40–49 years old, 26.1%, and 50–59 years old, 26.0%). The most proportion of the highest educational level is Junior college (33.8%) and Bachelor’s degree (34.5%). Most earned 3001–5000 RMB (36.8%) or 5001–10,000 RMB (30.5%) per month (1 USD = 6.77 RMB at the time of the survey).

**Table 1:** Participants’ demographic characteristics (n = 1523).

Variable	%(n)
Age	
18–29	22.1 (336)
30–39	25.9 (394)
40–49	26.1 (397)
50–59	26.0 (396)
Gender	
Male	51.5 (785)
Female	48.5 (738)
Education level	
Junior high school and below	12.0 (182)
Senior high school	17.2 (262)
Junior college	33.8 (515)
Bachelor degree	34.5 (526)
Postgraduate and above	2.5 (38)
Monthly income	
3000 or less	23.8 (363)
3001–5000	36.8 (560)
5001–10000	30.5 (464)
10,001–20,000	7.7 (117)
More than 20,000	1.2 (19)
Whether vaccinated or not	
yes	9.7 (148)
no	90.3 (1375)

### Descriptive statistical results of other variables

Data showed that the respondents generally agreed with compulsory vaccination management (*M*=3.5, *SD*=1.0). The study also measured participants' perceptions of the benefits of vaccination, the susceptibility, severity, and social threat (being quarantined and isolated) related to the infections. Generally, the public had a low level of perceived susceptibility (*M*=2.04, *SD*=1.3), moderate perceived severity (*M*=2.75, *SD*=1.5) and a high level of perceived benefit (*M*=3.52, *SD*=0.9). Additionally, they were concerned about COVID-19 infection-caused quarantine and isolation from others (*M*=3.13, *SD*=1.6).

Participants generally had a high level of nationalism (*M*=5.91, *SD*=1.2), a relatively strong belief in conspiracy theories (*M*=3.15, *SD*=1.16), and relatively moderate conspiratorial thinking (*M*=2.14, *SD*=0.9). Their science literacy level was moderate (*M*=5.48, *SD*=2.7).

Table 2 reported the highest level of the scales, mean, and standard deviation for each variable, as well as the results of the reliability and validity test of the questionnaire. Since the measurements of the four variables, such as perceived benefit, include only one item, the reliability and validity cannot be measured.

**Table 2:** Descriptive statistical results of variables (n = 1523).

Variables（scales）	*Mean*	*SD*	Cronbach’s *α*	KMO
Attitudes to mandatory vaccination (5)	3.50	1.0	0.70	0.67
Perceptions of Benefits (5)	3.52	0.9	/	/
Perceptions of Susceptibility (5)	2.04	1.3	/	/
Perceptions of Severity (5)	2.75	1.5	/	/
Perceptions of Being Quarantined (5)	3.13	1.6	/	/
Nationalism (7)	5.91	1.2	0.86	0.87
Conspiracy Theories Belief (5)	3.15	1.2	0.74	0.73
Conspiratorial Thinking (5)	2.14	0.9	0.90	0.87
Scientific Literacy (11)	5.48	2.7	0.83	0.89

### The association between risk and benefit perspections, political factors, conspiracy-related factors, science literacy and the attitude to mandatory vaccination

We examined the influencing factors of the public's attitude to mandatory vaccination of the COVID-19 vaccine. The first model examined demographic variables, including age, gender, education, monthly income, and COVID-19 vaccination status. Among them, only the monthly income was weakly correlated with the attitude to mandatory vaccination (*β* = 0.068, p <.01). However, after we added other variables in subsequent models, the effect was no longer significant.

The second model examined the relationship between risk and benefit perceptions and attitudes to mandatory vaccination. It included four independent variables: COVID-19 susceptibility perception, COVID-19 severity perception, social threat perception, and the perception of personal vaccination benefit. We found that all these four variables were, to varying degrees, positively correlated with mandatory vaccination attitudes and remained significant in Model 3. However, in Model 4, when we added conspiracy theory-related variables, factors other than the perceived benefit of vaccination no longer worked.

In the third step, we examined the nationalism level. The fourth model concentrated on the influence of COVID-19-related and vaccine-related conspiracy beliefs and people’s general conspiratorial thinking on their attitudes to mandatory vaccination. When we added these two types of factors to the models, their explanatory power increased by 20% of the variance. Nationalism (*β*=0.272, p <.000), belief in conspiracy theories (*β*=0.207, p <.000), and conspiratorial thinking (*β*=0.184, p <.000) strongly affected respondents' attitudes to mandatory vaccination.

The fifth model examined science literacy and its interaction with conspiracy beliefs and conspiratorial thinking. We found that science literacy had a minor, negative moderating effect on the association between conspiratorial thinking and the attitudes towards mandatory vaccination. The relationships are shown in Table 3.

**Table 3:** Hierarchical regression model of factors associated with attitudes to mandatory vaccination (n = 1,523).

**Variable**	**Attitudes to Mandatory Vaccination**				
	Model 1	Model 2	Model 3	Model 4	Model 5
	*β*	*β*	*β*	*β*	*β*
**Demography**					
Gender (ref. male) Female	−0.043	−0.018	−0.034	0.000	0.000
Age	0.024	0.027	0.009	0.007	0.002
Education (ref. Junior high school and below)					
Junior high school	0.025	0.026	0.019	0.089	0.039
Junior college	−0.046	−0.022	−0.042	−0.037	−0.014
Undergraduate degree	−0.034	0.006	−0.034	0.020	0.006
Postgraduate degree	−0.046	−0.040	−0.036	−0.161	−0.028
Income	0.068*	0.043	0.045	0.040	0.040
Whether vaccinated or not	−0.002	0.002	0.000	0.001	0.020
**Perceived Risks and Benefits**					
Perceptions of susceptibility		0.110***	0.119***	0.043	0.031
Perceptions of severity		0.078*	0.073*	0.049	0.052
Perceptions of being quarantined		0.131***	0.098**	0.052	0.055
Perceptions of personal benefits		0.175***	0.117***	0.148***	0.149***
**Nationalism**					
Nationalism			0.334***	0.297***	0.272***
**Conspiracy**					
Conspiracy beliefs				0.225***	0.207***
Conspiratorial thinking				0.178***	0.184***
**Literacy and Interactions**					
Scientific literacy					0.016
Science literacy × Conspiracy beliefs					−0.043
Science literacy × Conspiratorial thinking					−0.070**
**Model statistics**					
Adjusted *R^2^*	0.006	0.111	0.217	0.320	0.328
Δ*R^2^*	0.011	0.107	0.106	0.103	0.009
Δ*F*	2.172*	45.822***	205.643***	114.860***	6.860***

Notes. *p <.05. **p <.01. ***p <.001.

In general, the results supported H3 and H5 but rejected H1, H2, H4 and H8. Regarding H6 and H7 on the impact of conspiracy theory beliefs and conspiratorial thinking on the mandatory vaccination against COVID-19, the results showed exactly the opposite directions. The results were reported in Table 4 (mandatory vaccination was abbreviated as MV in the table). We will discuss the results in the following section.

**Table 4:** Testing results of hypotheses.

Hypotheses	Testing results
H1 The higher the perceived susceptibility, the greater support for MV.	rejected
H2 The higher the perceived severity, the greater support for MV.	rejected
H3 The higher the perceived benefit, the greater support for MV.	supported
H4 The higher the risk of being quarantined, the greater support for MV.	rejected
H5 The higher nationalism, the greater support for MV.	supported
H6 The firmer conspiracy beliefs, the less support for MV.	reversely supported
H7 The stronger conspiratorial thinking, the less support for MV.	reversely supported
H8 The higher the science literacy, the greater support for MV.	rejected

**Fig. 1:**
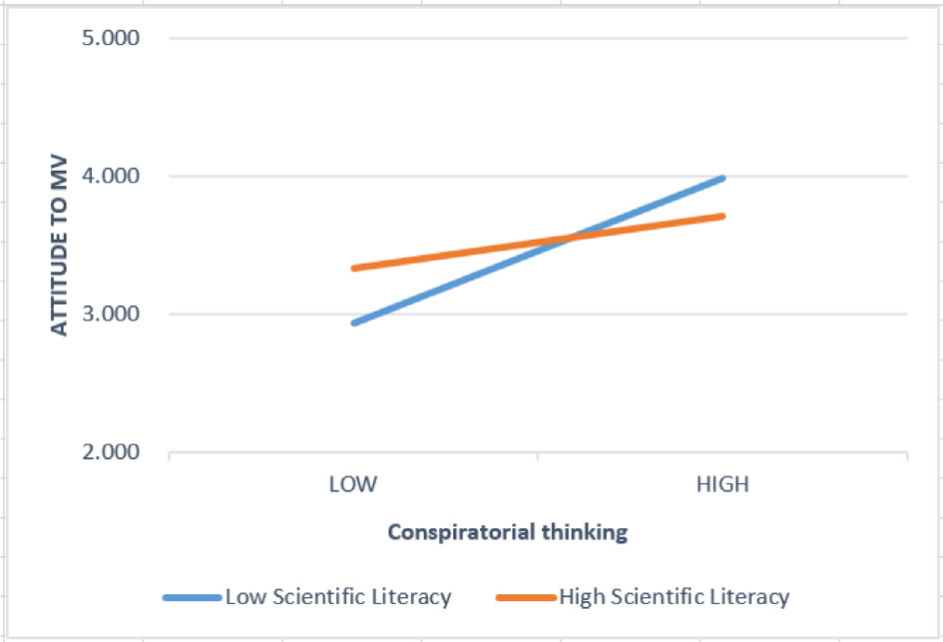
Moderating effect of scientific literacy.

For Q1, science literacy only negatively regulated the relationship between conspiratorial thinking and mandatory vaccination. Fig. 1 demonstrated the moderation effect of science literacy in the association between conspiratorial thinking and attitude to mandatory vaccination. It showed that compared with those with lower science literacy (-1*SD*), among those with higher science literacy (+1*SD*), the positive influence of conspiratorial thinking in attitude increased more slowly.

## Discussion and conclusions

The findings offered fresh evidence of the relationship between the attitude towards mandatory vaccination and the Chinese public’s politicized beliefs which includes their nationalism, faith in conspiracy theories in the context of COVID-19 pandemic as well as the general tendency to accept conspiratorial explanations. First, the results differed from many studies in that all demographic variables – age, education level, gender, income, and whether vaccinated – cannot predict the attitude to vaccine mandate [7], [16], [53], [54]. The fact that no demographic element influenced the Chinese attitude to a vaccination policy and the very low variance explained by Model 1 may show that the Chinese public did not consider vaccination very personally relevant.

As indicated in Models 2 through 4, although initially, risk and benefit perceptions were all significant determinants for the attitude to mandatory vaccination, the significance of most of them disappeared after conspiracy beliefs and conspiratorial thinking were added to the model. Only the perceived benefit of the exemption of infection was still a significant determinant. As a result, our H1, H2 and H4 were rejected and only H3 was supported. As discussed below, the role of personal risk perceptions in forming the Chinese public’s attitude to vaccine mandates might be overridden by conspiracy beliefs and conspiratorial thinking. The reason for the relatively stable association between the perceived benefit of vaccination and the attitude to compulsory vaccination was unknown, but we suspect that, unlike personal risk feelings, which are more likely to be individualized, the benefit perception could be more strongly related to people’s perception of their relationship with the state.

In Model 3, which tested H5, the addition of nationalism not only brought a strong determinant for the attitude to mandatory vaccination but also dramatically increased the model’s explanatory power. The higher respondents had a nationalism score, the more likely they would endorse compulsory vaccination. Consisting with previous studies, the Chinese public’s attitude to mandatory vaccination was politicized and also reflected a statism value [30], [32], [55]. Another point worth mentioning is in this model, the addition of nationalism didn’t change the statistical significance of risk and benefit perceptions and only had minor impacts on their coefficients. It may mean that nationalism jointly functioned with individual perceptions of risks and benefits.

Model 4, which tested H6 and H7, added conspiracy beliefs and conspiratorial thinking, and its explanatory power was significantly increased. However, it brought contrary results to previous studies, which often showed that the conspiracy belief was linked to the opposition to masks and vaccines [56]. In our research, the faith in a batch of conspiracy theories – covering both the pandemic origin and vaccine manipulation – was positively rather than negatively associated with the attitude to vaccination mandates. In addition, conspiratorial thinking, which felt actions of the government incomprehensible and hence should have encouraged the rejection of vaccination required by the government [42], [57], instead positively predicted the attitude to mandatory vaccination in our study. The results were in opposite directions to our original hypotheses. The likely reasons for the contradictory results may lie in the Chinese public’s strong statism. Because various conspiracy theories claiming foreign origin of SARS-CoV-2 or fabrication of vaccine safety data all imply vicious intention, people who believed these theories thus felt greater need for the stronger protection from the state, hence they would follow the government’s compulsory vaccination policies. Moreover, since the behavior of the government is incomprehensible – as measured in conspiratorial thinking – the ordinary people should simply follow its rules. In terms of the relationship between key variables, we may understand the disappearance of the risk perceptions' statistical significance as this: the risk our respondents felt is primarily not from the natural infection of SARS-CoV-2 but more from the conspiracies implemented by evil forces.

In Model 5, science literacy didn’t predict attitudes to mandatory vaccination. Our H8 was not supported. The addition of science literacy didn’t remarkably improve the explanatory power of the whole model, and neither did it have a major impact on other statistically significant determinants. The measure’s moderation role was weak. It can only weakly moderate the positive association between conspiratorial thinking and the attitude to mandatory vaccination. This might be caused by the stronger political imprint in the Chinese mind, which shaped their views more than scientific knowledge or analytical reasoning covered by the measure of science literacy. However, higher science literacy indeed weakened the influences of conspiratorial thinking, though the overall role of science literacy is minimal in influencing people’s attitude to mandatory vaccination.

As a whole, our study reveals a sharply distinct scenario regarding the public attitude to vaccination policies. The politicized determinants – nationalism, conspiracy beliefs, and conspiratorial thinking – played a vital explanatory role while most of typical predictors – demographic factors and individual risk perceptions – were not significantly associated with the attitude to mandatory vaccination. While the situation may result from China's successful quarantine policies, which dramatically reduced the COVID-related hazards visible to the public, it may also be caused by the country's powerful mobilizational and organizational capacity. The weaker function of personal risk perceptions can also be rooted in the collectivist orientation in the Chinese sociopolitical culture. However, the attitude was also linked to the Chinese public's conspiracy belief and entrenched conspiratorial thinking, which can hardly be rational. No matter how conspiracy beliefs and conspiratorial thinking can strongly predict attitudes to vaccination policies, we cannot rely on them to increase people's vaccination intentions and improve the nation's overall vaccination coverage.

The current study highlights the politicized nature of the factors influencing people's attitudes to vaccines and vaccination policies in China, which were previously neglected. As China urgently needs to improve its booster vaccine coverage, more studies are required to explore how to utilize our findings to fulfill the goal.

Our effort to investigate politicized determinants of vaccination and other health behaviors can go beyond the COVID-19 setting. In this highly politically polarized world, partisanship is found to have influenced many public health measures and behaviors, such as reducing soda uptake to fight diabetes and obesity [57]. China shouldn't be an exception. Future studies can focus on those behaviors that can be politicized in China, such as accepting domestically developed innovative drugs and therapies.

For practical implications, our study demonstrates that politicized factors triumphed over individual risk perceptions to dominate the Chinese public’s attitude to vaccination policies. But this doesn’t mean personal risk and benefit perceptions should be overlooked during the campaign to mobilize Chinese people to vaccinate. Instead, we should strengthen the information to help individuals make rational decisions. However, such an edification process can be done in a nationalistic tone to improve the persuasion effect in China [30]. With more studies exploring the politicized aspect of Chinese people’s public health behaviors, indoctrinating medical information through nationalistic or collectivistic messages might be adoptable in other health scenarios.

Finally, our research has some limitations and is worth further improvement. First, this study was based on a cross-sectional survey, which reminds us to be cautious in reaching any causational conclusion. Second, our April 2021 data are relatively older. However, we don't think these limitations reduced the validity of this study. The politicization of the Chinese public's attitude towards and intention for COVID-19 vaccination is visible in many studies [30], [31], [32], [36], [58]. Follow-up research might address this limitation with new data and methods while exploring the political aspects of Chinese people’s health behaviors and attitudes.

## Funding

This study is supported by Key Project “Study on the permanent mechanism of communicating scientific spirit and professionalism in the digital era” of the National Social Science Foundation of China (No.21AZD013); Postgraduate Research & Practice Innovation Program of Jiangsu Province“A study on the Weibo framework of ‘great anti-epidemic spirit’ under the new situation of COVID-19 pandemic preventions”(KYCX22_3168); and The 2022 Postgraduate Science Popularization Capability Promotion Program of China Association for Science and Technology “Research on the Evaluation and Guidance Mechanism of Scientists' Participation in Science Popularization Creation -- Taking COVID-19 Science Popularization as an Example” (KXYJS2022015).

**Institutional Review Board Statement:** The study was conducted according to the guidelines of the Declaration of Helsinki and approved by the School of Communication, Soochow University.

**Informed Consent Statement:** Informed consent was obtained from all subjects involved in the study.

**Data Availability Statement:** Materials and anonymous data are available from the authors by request.

## CRediT authorship contribution statement

**Ruifen Zhang:** Conceptualization, Methodology, Writing – original draft. **Jun Yan:** Conceptualization, Writing – review & editing. **Hepeng Jia:** Conceptualization, Methodology, Writing – original draft, Writing – review & editing. **Xi Luo:** Methodology, Writing – review & editing.

## Declaration of Competing Interest

The authors declare the following financial interests/personal relationships which may be considered as potential competing interests: Hepeng Jia, Ruifen Zhang reports financial support was provided by National Social Science Foundation of China, the Postgraduate Research & Practice Innovation Program of Jiangsu Province, and the 2022 Postgraduate Science Popularization Capability Promotion Program of China Association for Science and Technology.

## Figures and Tables

**Table:** Specific questions of the scales.

Attitudes towards mandatory vaccination	
1	Compared with people who have been vaccinated with the COVID-19 vaccine, those who have not should have less freedom of movement.
2	It is reasonable and legal for the government to force people to vaccinate against COVID-19.
3	The public has the right to choose whether to vaccinate themselves with COVID-19 vaccine.
4	When necessary, the government has the right to compel individuals to show COVID-19 vaccination records in public places.
Perception of Benefits	
	Inoculation of COVID-19 vaccine will prevent me from being infected with COVID-19.
Perception of Susceptibility	
	There is a good chance that I will have COVID-19.
Perception of Severity	
	I am worried that I will die of COVID-19.
Perception of the threat of being quarantined or isolated	
	I am worried that I will be quarantined or isolated by people around me because I have Covid-19 or become a suspected case.
Nationalism	
1	I prefer to be a Chinese citizen rather than a citizen of other countries.
2	My country is better than most other countries.
3	We should support our government even if it is wrong.
4	China outperformed other countries in controlling COVID-19.
5	China excelled other countries in protecting its citizens from the effects of the epidemic.
6	After the pandemic, China's economic recovery will be faster than that of any other country.
Participants’ Belief in Conspiracy theories	
1	The coronavirus came out of a foreign military laboratory.
2	The coronavirus originated from a virus research institute in Wuhan, China.
3	Pharmaceutical companies often fabricate vaccine effectiveness data.
4	5G technology helps spread the coronavirus.
Participants’ Conspiratorial Thinking	
1	The inside story of many essential things in the world is kept from the public.
2	Officials often do not tell us the real motives behind their decisions.
3	Government agencies closely monitor all citizens.
4	Incidents that appear to be unconnected are often the result of covert activities.
5	Some secret organizations have a significant influence on political decision-making.
Scientific literacy	
1	Two scientists wanted to know if a drug to treat high blood pressure was effective. The first scientist gave the drug to 1,000 patients with high blood pressure and then observed how many patients had a decrease in blood pressure; The second scientist divided the patients into two groups, the first group of 500 hypertensive patients taking the drug and the other group of 500 patients not taking the drug, to see how the blood pressure dropped in the two groups. **Which of the first and second scientists tested the drug’s effect more effectively?**
Doctors tell a couple that because they share the same pathological genes if they have a child, the chance of that child developing a genetic disease is 1 in 4. The following statement, whether you think it is correct or not.	
2	If they have three children, none of the three children will get a genetic disease.
3	If their first child has a genetic disease, the next three children will not have it.
4	If the first three children are healthy, then the fourth child must have a genetic disease.
5	Their children can all get genetic diseases.
Do you think the following statement is correct?	
1	Coronaviruses can cause SARS and pneumonia but not colds.
2	Electrons are smaller than atoms.
3	The mother's genes determine whether the fetus is a boy or a girl.
4	Lasers are produced by converging sound waves.
5	Antibiotics (such as penicillin, streptomycin, or cephalosporin) kill both bacteria and viruses.
6	If genetically modified fruits are eaten, a person's genes may change.

## Data Availability

Data will be made available on request.
